# Intravagal parathyroid adenomas: Case report and literature review

**DOI:** 10.1002/ccr3.2855

**Published:** 2020-04-13

**Authors:** Marna A. List, Brian J. Boyce, Peter T. Dziegielewski

**Affiliations:** ^1^ College of Medicine University of Florida Gainesville Florida; ^2^ Department of Otolaryngology University of Florida Gainesville Florida; ^3^ Department of Otolaryngology‐Head and Neck Surgery Emory University Atlanta Georgia; ^4^ UF Health Cancer Center UF Health Shands Hospital Gainesville Florida

**Keywords:** adenoma, hyperparathyroidism, intravagal, parathyroid, vagus nerve

## Abstract

Intravagal parathyroid adenomas remain an exceedingly rare diagnosis; however, their true incidence may be higher than currently known. It is important to keep intravagal sites within the list of potential ectopic locations of parathyroid adenomas.

## Graphical abstract

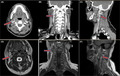

## 1 INTRODUCTION

During embryogenesis, parathyroid glands develop from the third and fourth pharyngeal pouches and descend to their respective destinations at the inferior and superior locations on the dorsum of the thyroid.^1^ A disruption in this migration can lead to ectopic parathyroid tissue anywhere along this path or within adjacent tissues. Ectopic parathyroid tissue is a well‐recognized phenomenon and has been documented in locations from the carotid sheath to the middle mediastinum.^2^ It is important to recognize the potential for abnormally located parathyroid tissue as parathyroid adenomas are treated surgically.

While ectopic parathyroid adenomas have been well described, intravagal parathyroid adenomas are rare and have only been documented in the literature in 12 previous cases.^3^, ^4^, ^5^, ^6^, ^7^, ^8^, ^9^, ^10^ Here, we describe the identification, management, and postsurgical outcomes of two additional cases of parathyroid adenomas within the vagus nerve.

## 2 HIGHLIGHTED CASES

**Figure 1:**
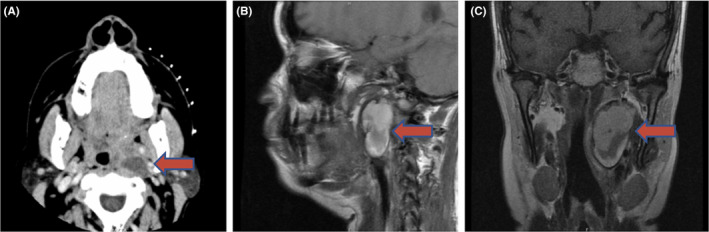
A, Transverse CT demonstrating an ovoid heterogeneous mass with partial occlusion of the oropharynx. B, Sagittal T2‐weighted magnetic resonance scan showing the anterior displacement of the carotid arteries due to the mass effect. C, Coronal T1‐weighted magnetic resonance scan revealing the location of the mass and the effect on surrounding structures

Patient 1 is a 52‐year‐old female who presented to clinic for evaluation of a neck mass and associated dysphonia, dysphagia, persistent cough, and left neck, ear, and jaw pain for three months. Physical examination revealed a bulging left tonsil displaced medially, and flexible laryngoscopy showed left true vocal fold paralysis. Her serum calcium level was 12.9 mg/dL. CT and MRI revealed a 2.3 cm parapharyngeal mass within the left carotid sheath displacing both the internal and external carotid arteries anteriorly (Figure 1). A CT‐guided fine‐needle aspiration was performed and was originally interpreted as thyroid tissue due to thin colloid with groups of follicular‐type cells in the sample. Resection of the mass without capsule disruption was performed via a transoral approach and revealed a 5 × 3 cm mass, though unusually large surgical pathology confirmed a benign parathyroid adenoma without nuclear atypica, foci of necrosis, or increased mitotic activity. The mass was inferior to the skull base and superior to the carotid bifurcation. Her immediate postoperative calcium level was at 9.0 mg/dL. On postoperative day 3, mild ptosis and anisocoria were noted of the left eye as well as left‐sided mydriasis, consistent with a mild Horner's syndrome. Tongue protrusion had slight leftward deviation. She was discharged on postoperative day 5. Tongue weakness resolved by 1 month post‐op, and Horner's syndrome resolved by 1 year. Left true vocal fold never regained function and was stable from its pretreatment state.

**Figure 2:**
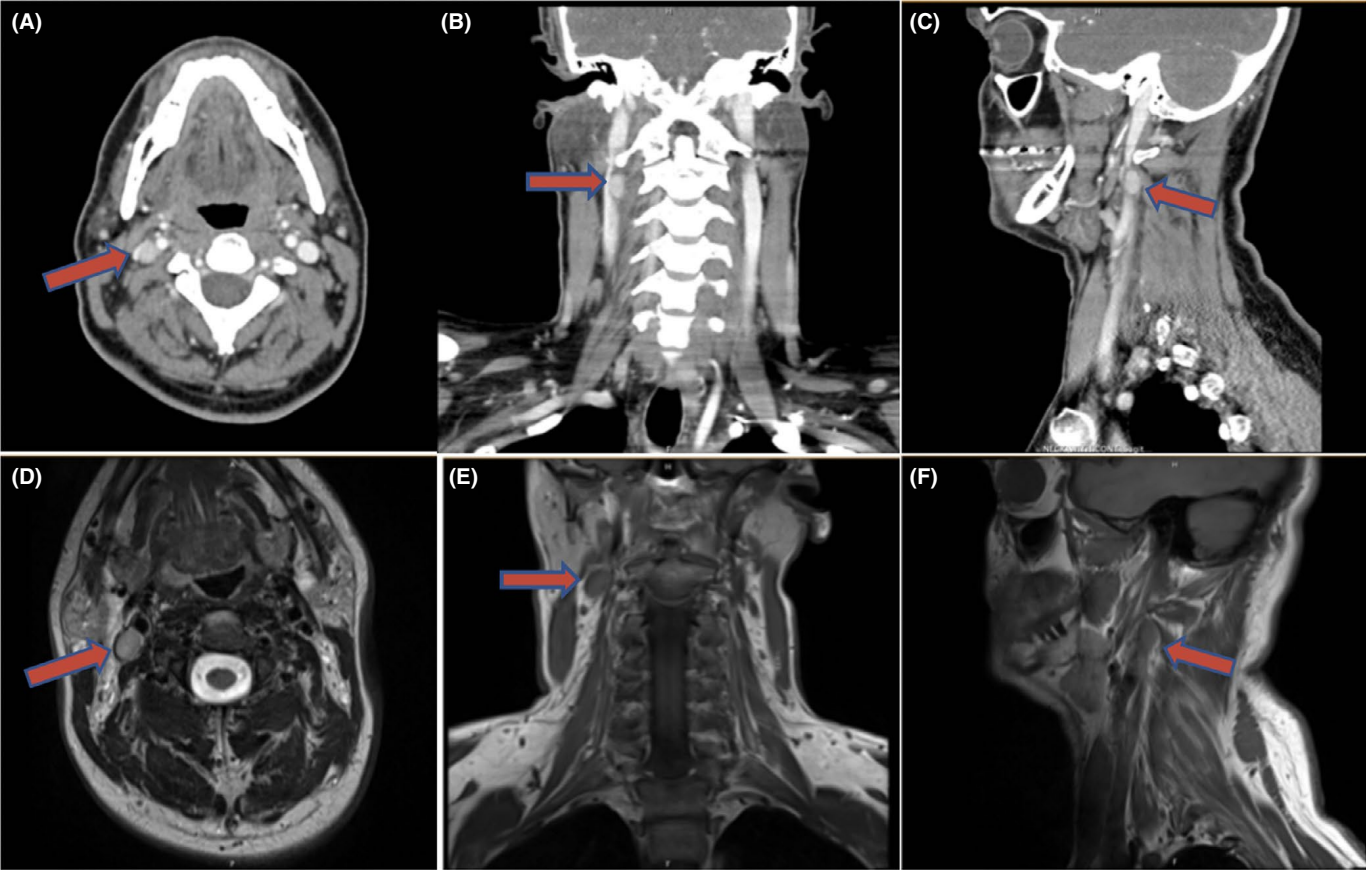
Corresponding CT and MRI images of intravagal parathyroid adenoma in Patient 2. A/D, Transverse cut demonstrating an ovoid mass at the level of the carotid bifurcation. B/E, Coronal view showing the relationship of the mass to the skull base. C/F, Sagittal plane demonstrating an ovoid mass within the carotid sheath

**Figure 3:**
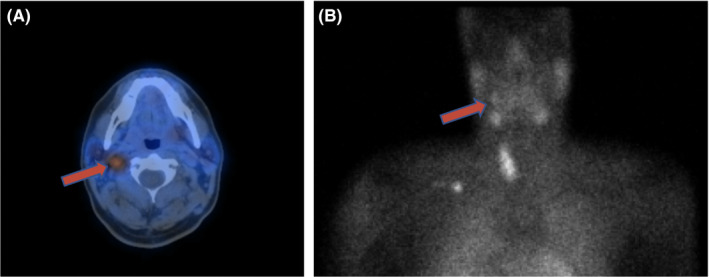
A, Fusion SPECT sestamibi parathyroid scintigraphy demonstrating uptake in the right carotid sheath. B, Anterior global image of sestamibi parathyroid scintigraphy showing focal uptake in the right carotid sheath and physiologic uptake in the bilateral parotid glands, bilateral submandibular glands, and the remaining right thyroid lobe

Patient 2 is a 34‐year‐old male with known hyperparathyroidism who presented to clinic for evaluation of persistent hypercalcemia. Three months prior, he underwent a left thyroid lobectomy and failed left parathyroid exploration after imaging showed growth of a left thyroid nodule. Surgical pathology revealed a 3 cm minimally invasive follicular thyroid carcinoma, a separate 1 mm focus of papillary thyroid carcinoma, and non‐hypercellular superior and inferior left parathyroid glands. On presentation, he was found to have calcium at 12 mg/dL and had symptoms of fatigue, mild dysphagia, and dysphonia. Physical examination was unremarkable aside from his surgical scar. CT neck with contrast showed an enhancing nodule at the level of the right carotid bifurcation lying medial to the jugular vein and posterior to the carotid artery near the skull base. The washout characteristics of this nodule were consistent with a parathyroid adenoma, and an MRI confirmed these findings (Figure 2). Nuclear medicine parathyroid scan using IV sestamibi revealed focal uptake in the right carotid sheath corresponding to this nodule (Figure 3). Resection was performed via a transcervical approach, and a mass measuring 0.8 × 0.6 × 0.4 cm was dissected out from the vagus sheath in toto. Frozen section was consistent with hypercellular parathyroid gland. Intraoperative PTH increased to 365 pg/mL further confirming the mass as parathyroid tissue. Postoperative PTH dropped to 27 pg/mL, and postoperative calcium normalized at 8.3 mg/dL. A completion thyroidectomy was also performed. The patient was discharged the next day with a weak voice but no other deficits. Three months later his vocal cord was paretic, but he had a strong voice.

## 3 DISCUSSION

Intravagal parathyroid adenomas have only been reported 12 times in the literature. Lack et al (1988) suggested that the incidence may be higher than currently known. His study examined the vagus nerves of 32 deceased infants under 1‐year‐old and found that 6% contained parathyroid cells, confirmed by chromogranin and PTH staining. This finding demonstrates that there may be an impressive subset of the population that is living with asymptomatic ectopic parathyroid tissue within the vagus nerve. This unusual location of parathyroid tissue was postulated to arise due to the close embryologic origins of both the vagus nerve and the parathyroid glands.^11^

Both patients presented in this case series underwent different workups due to the differences in their presentations and relevant past medical histories. Patient 1 presented with symptoms due to mass effect and was worked up with a biopsy because of the unclear nature of the mass. It was removed via a careful transoral approach. Due to extensive dissection at the skull base, she had temporary cranial nerve deficits but recovered to baseline. This can certainly be expected with a transoral approach to the parapharyngeal space.^12^ Patient 2 underwent parathyroid‐specific imaging as an ectopic parathyroid gland was expected. His approach was transcervical, and his only sequalae was vocal cord paralysis, which is recovering well. Both Patient 1 and 2 normalized their calcium and PTH levels postoperatively. Though the workup and surgical approaches varied, they were both equally appropriate given the clinical presentation and location of the mass.

The current known cases of intravagal parathyroid adenomas (Table 1) follow a similar sex ratio trend to the true incidence of parathyroid adenomas. Particularly, the ratio of female to male in this series is 2.5:1, mirroring the 2‐3:1 female‐to‐male ratio of primary hyperparathyroidism in the population.^13^ The average age at presentation is 45 years, and all patients present with hypercalcemia. Preoperative imaging studies included all possible modalities as intravagal adenomas are challenging to diagnose on imaging. Eight (57%) patients contained left‐sided adenomas. There was a notable predominance of adenomas located above or at the carotid bifurcation (7 and 5, respectively) compared to only 2 found below the carotid bifurcation. The sizes of the lesions varied from 2 to 50 mm at greatest diameter. All patients were treated surgically, and surgical pathology was universally found to show hypercellular parathyroid tissue. Two (14%) developed hypocalcemia following the surgery. Four (28%) experienced postoperative transient laryngeal nerve palsies while Patient 1 in this study presented with vocal cord paralysis.

**Table 1:** Compares the case details of two adults diagnosed with intravagal parathyroid adenomas at the University of Florida Shands Hospital compared to the case details of all additional documented intravagal parathyroid adenomas in the English literature

	Patient 1				Patient 2		Patient 3		Patient 4	Patient 5	Patient 6	Patient 7	Patient 8	Patient 9	Patient 10	Patient 11	Patient 12	Patient 13	Patient 14
Author y	Dz 2019 (this study)				Dz 2019 (this study)		O’Neil et al ^10^ 2019		Daruwalla et al ^9^ 2015	Chan et al ^8^ 2003	Chan et al ^8^ 2003	Chan et al ^8^ 2003	Chan et al ^8^ 2003	Pawlik et al ^7^ 1999	Pawlik et al ^7^ 1999	Buell et al ^6^ 1995	Doppman et al ^5^ 1994	Takimoto et al ^4^ 1989	Reiling et al ^3^ 1972
Sex	Female				Male		Male		Female	Female	Female	Female	Female	Male	Male	Female	Female	Female	Female
Age (y)	52			34			43		17	‐	‐	‐	‐	48	43	‐	40	79	55
Presenting symptoms	Dysphonia, dysphagia, left ear and jaw pain, cough				Fatigue, mild dysphagia, dysphonia		None		Fatigue, nephrolithiasis	‐	‐	‐	‐	Fatigue, muscle cramps, weakness, bone pain	Nephrolithiasis, fatigue, forgetfulness, abdominal discomfort	‐	Fatigue, muscle weakness, constipation, nausea	Enlarging left sided neck mass	‐
Presenting serum PTH	134				121		NA		12.1 pmol/L	Elevated	Elevated	Elevated	Elevated	1000 pg/ml	98 pg/ml	‐	74 pg/ml	2.14 ng/ml	‐
Presenting serum calcium	12.9 mg/dL		12 mg/dL				NA		2.81 mmol/L	Elevated	Elevated	Elevated	Elevated	11 mg/dl	12.3 mg/dl	‐	2.5‐3.12 mmol/l	6.4 mEq/l	‐
Imaging history	US, CT, MRI				US, CT, MRI, SS		US, CT, Dual tracer^a^, SG		US, SS	US, SA	US, SA, SVS	SA, SVS	CT, MRI, SG	CT, SS, SVS	Left neck US, SS, SVS	US, SA, MRI, SVS, CT, SG	US, MRI, CT, SG, arteriography, SVS	US, CT, SG	US, CT, SG
Size of lesion	5 x 3 cm				0.8 x 0.6 x 0.4 cm		14 mm		7 mm	13 x 8 x 7 mm	6 x 5 x 2 mm	12 x 8 x 8 mm	20 x 8 x 6 mm	1.0 x 1.2 cm	1.0 x 1.4 cm	‐	1.2 x 0.8 cm	1.7 x 2.5 cm	0.5 x 0.8 cm
Location of lesion	Left VN above level of CB	Right VN at the CB				Left VN above level of CB		Left VN at level of CB		Left VN above level of CB	Right VN above level of CB	Right VN above level of CB	Left VN below level of CB	Left VN at level of CB	Left VN at level of CB	Right VN above level of CB	Right VN above level of CB	Left VN below level of CB	Right VN at level of CB
Approach	Transoral approach surgical resection	Skull base approach surgical resection					Level I‐II selective neck dissection and surgical excision		Left‐sided parathyroid exploration, left cervical thymectomy	Left oblique cervical approach resection	Right oblique cervical approach	Right oblique cervical approach	Left oblique cervical approach	Left lateral cervical approach and excision	Left lateral cervical approach and excision	‐	Anterolateral cervical approach and excision	‐	Right lateral cervical approach and excision
Surgical Pathology	Hypercellular parathyroid adenoma				Hypercellular parathyroid adenoma		Hypercellular parathyroid adenoma		Parathyroid tissue	Hypercellular parathyroid adenoma	Hypercellular parathyroid adenoma	Hypercellular parathyroid adenoma	Hypercellular parathyroid adenoma	Hypercellular parathyroid adenoma	Hypercellular parathyroid adenoma	Hypercellular parathyroid adenoma	Hypercellular parathyroid adenoma	Parathyroid adenoma with cystic degeneration	Hypercellular parathyroid adenoma
Post‐op serum calcium	8 mg/dL				(ionized) 1.45 mmol/L		Normalized		2.31 pmol/L	normalized	normalized	normalized	normalized	9.7 mg/dl	5.0 mg/dl	‐	‐	5.6 mEq/l	normalized
Post‐op symptoms	Left true vocal fold paralysis, left tongue deviation, Horner syndrome			None			Transient recurrent laryngeal nerve paresis		None	‐	‐	‐	‐	None	Transient left vocal fold paresis	‐	Transient hypocalcemia and transient recurrent laryngeal nerve palsy	Transient left recurrent laryngeal nerve palsy	‐

Abbreviations: CB, carotid body; SA, selective angiography; SG, scintigraphy; SS, 99m Tc sestamibi scan; SVS, selective venous sampling; VN, vagus nerve.

^a^ Pertechnetate/sestamibi subtraction.

Single‐gland parathyroid adenomas are the leading cause of primary hyperparathyroidism contributing to 80%‐85% of cases.^14^ It is important to be able to localize the source of hyperparathyroidism prior to surgical intervention, though this proves to be challenging. Imaging modalities such as 4D CT scanning and SPECT scans can be useful in identifying an abnormally located parathyroid glands. O’Neil et al reported success in using a gamma probe intraoperatively to help identity an intravagal parathyroid adenoma.^10^ The use of a gamma probe intraoperatively as an additional tool to help identify parathyroid tissue was described by Norman et al who treated patients with IV Tc99 sestamibi prior to surgery and then used the probe to localize parathyroid tissue allowing for a more minimally invasive approach.^15^ The use of preoperative imaging and intraoperative localization may prove to aid in the identification of ectopic parathyroid adenomas.

Surgical approach for intravagal parathyroid adenoma is dependent on the location and size of the lesion. In the literature, the dominant approach is transcervical with 12 out of 14 (85.7%) patients, while transoral and skull‐based approaches constituted 1 out of 14 (7.1%) cases each. The approach for intravagal parathyroid adenomas has not been studied in itself due to the low volume of cases; however, there is literature describing approaches used for other intravagal pathology such as shwannoma and paraganglioma. The most common approach for intravagal tumors is transcervical either with or without parotidectomy or mandibulotomy.^16^, ^17^ Other approaches include transoral, and more recently, transoral robotic surgery (TORS) has been explored in its utility to excise neurogenic neoplasms from the parapharyngeal space. In select cases, TORS may provide a solution to the instrumentation and visual limitations of the transoral approach to the parapharyngeal space.^18^ Due to the location of these tumors within or around the vagus nerve in relation to other vital structures, there are inherent risks to surgery. Postoperative morbidity in patients undergoing vagus nerve surgery includes recurrent laryngeal nerve palsy or Horner's syndrome due to manipulation or sacrificing of the vagus. In addition to careful dissection and selecting the appropriate approach, there may be a role for neural integrity monitor (NIM) electromyogram (EMG) tube placement in order to reduce postsurgical morbidity. Use of a NIM‐EMG tube has been shown to aid in nerve preservation during the removal of vagal nerve schwanommas and decrease postoperative morbidity.^19^

## 4 CONCLUSION

Intravagal parathyroid adenomas remain an exceedingly rare diagnosis; however, their true incidence may be higher than currently known. It is important to keep intravagal sites within the list of potential ectopic locations of parathyroid adenomas during the workup for primary or persistent hyperparathyroidism.

## CONFLICT OF INTEREST

None declared.

## AUTHOR CONTRIBUTIONS

MAL: involved in design and conception, data collection, data analysis, data interpretation, and manuscript drafting; BJB: involved in design and conception, data interpretation, and manuscript editing; PTD: involved in design and conception, data interpretation, manuscript editing, and supervision.

## Data Availability

All data generated or analyzed during this study are included in this published article.
